# Development of epoxy-based sandwich composite panel with hollow glass microspheres/clay hybrid core and banana fiber facesheet for structural applications

**DOI:** 10.1016/j.heliyon.2024.e30428

**Published:** 2024-04-27

**Authors:** Ayodele Abraham Ajayi, Mohan Turup Pandurangan, Kanny Krishnan

**Affiliations:** Composite Research Group, Department of Mechanical Engineering, Durban University of Technology, South Africa

**Keywords:** Sandwich, Composites, Core, Facesheet, HGM, Nanoclay, Epoxy

## Abstract

This study focuses on improving the mechanical properties of sandwich composites by developing epoxy-based sandwich composite panels with hollow glass microspheres/nanoclay hybrid core and banana fiber facesheets for structural applications. The mechanical performance of sandwich composite panels made with hollow glass microspheres (HGM)/nanoclay hybrid core with banana fibers face-sheet composites panel is investigated in this work. The HGM content of the core was varied from 1 wt% to 3 wt% in the sandwich composites panel, while the nanoclay content of the core was varied from 1 wt% to 5 wt% in each of the HGM-filled series of the sandwich composite panel, these sandwich composite panels were fabricated using a conventional resin casting method. In this investigation, the mechanical, water absorption, and buoyancy behavior are thoroughly studied and the findings revealed better improvement at the sandwich composites with hybrid core formulation with banana fiber facesheets than the sandwich composites without hybrid core formulation. This demonstrates that banana fiber with epoxy resin has a limited amount of strength when used without a hybrid core but delivers better performance when HGM and clay particles are mixed as the hybrid core because of excellent interfacial adhesion between the hybrid core and the matrix. The improved mechanical properties could suggest that this material may be suitable for application in industries where sandwich structures that are lightweight with good mechanical properties are required. This study showed a new area of sandwich structure development by enhancing mechanical properties using hybrid core and banana fibers.

## 1 Introduction

Sandwich composite structures (SCS) are structures made up of three layers: a low-density core, a thin skin, or a facesheet layer bonded to each side of the core. SCS are employed in applications that require a high structural rigidity while remaining lightweight and they are important materials in a wide range of engineering applications, including aerospace, automotive, marine, and wind energy [[1], [2], [3], [4]]. Due to their unique design, lightweight, high strength, and stiffness of the sandwich core composite structure, as well as their significance in the engineering technological fields of aerospace, automotive, marine, and construction, sandwich composite material structures are highly important to study. Sandwich structure principles have been used for more than two decades using various processing methods. Polymeric foams have been the core material of sandwich structures in the past [5,6]. The advantages of a cellular polymer foam core over metallic or metallic foam cores are low cost and ease of manufacture. For space applications, sandwich constructions made of cellular polymer foam offer low-velocity impact, blast mitigation, crashworthiness, and shock wave absorption capabilities. Various polymers used to develop cores in sandwich structures include polyurethane [7], poly(vinyl chloride) [8] ], polystyrene [9], styrene-acrylonitrile [10], and other matrices. Furthermore, mechanical performance on sandwich composites consisting of syntactic foam filled with hollow glass microsphere in an epoxy resin matrix as the core material and hybrid kenaf/glass fibers face-sheet based composites panel has been studied [11] but using HGM/nanoclay as hybrid core and banana facesheet has not been explored which is the focus of the current study. Sandwich composite facesheets bear the different bending stresses which act on the panel. The reinforcement is provided by the core, which ensures enough shear stiffness with low specific weight to absorb applied shear pressures. As a skin, several facesheets have been utilized, ranging from natural to glass fibers such as sisal, jute, kenaf, flax, glass, and paper [[12], [13], [14], [15], [16], [17]]. Studies have explored utilizing natural fibers in the fabrication of epoxy-based composite structures [18,19]. Studies have confirmed that using flax fibers as a facesheet of sandwich structures can enhance their flexural properties, the results reported contribute to the sustainability of sandwich composites with the usage of natural fibers [20]. Also, Wagas et al. hybridized kenaf and flax fiber as facesheet of sandwich composite structures and studied their impact behavior and it was discovered from their findings that the hybridized facesheet enhanced better energy absorption rate than the glass fiber facesheet [21].Benjamin et al. [22]also studied the flexural behavior of sandwich structures made with hemp fiber facesheet to bending stress and it was reported from their findings that facesheet made with hemp fibers can enhance the bending strength of sandwich panel [22].Natural fiber composites hereditably perform poorly in the presence of moisture [23], affecting the morphology of the natural fiber [24], crack propagation [25], and sliding surface reaction [25].Even with these natural fiber benefits, manufacturing polymer composites with natural fibers is significantly hampered by the incompatibility of the polymer matrix and fiber surface [26]. However, natural fibers can undergo chemical treatment to change the network structure by breaking hydrogen bonds and roughening the surface. In this current work, 5%NaOH chemical treatment of fibers was selected because it has proven to be effective according to previous research [27]. In the past, banana fiber was largely utilized to create ropes, carpets, and other composite materials. But as people's awareness of the environment and demand for eco-friendly fabrics has increased, banana fiber has gained recognition for all of its advantages and is increasingly being used in a variety of other industries, such as home furnishings, aerospace, automotive, and apparel sectors [28,29]. Research indicates that the use of banana fibers as a sustainable and environmentally friendly reinforcing material in epoxy-based composites has increased [30].Therefore, using banana fiber as facesheets with a hybrid core is one of the most effective methods of overcoming the inferior mechanical properties of natural fibers [31].However, a variety of core materials have been previously used for sandwich composite panels but they still lack sufficient stiffness and toughness properties. These drawbacks limit their applications, particularly for aerospace and other critical engineering applications. Nonetheless, hybrid cores are favored due to their high degree of bending stiffness, strong thermal insulation, low-density qualities, and reduced weight [32,33]. Using hybrid-filled foam as the core material which contains hollow glass microspheres (HGM) and nanoclay reduces weight in maritime applications while also improving the performance of the sandwich construction [32,[34], [35], [36]] because HGM is made out of borosilicate-soda lime glass blend formulation which provides benefits of low density, high heat, and chemical resistance while nanoclay consist of nanoparticles of layered mineral silicates that enhances the bending stiffness of materials [37,38]. Also, hollow glass microspheres (HGM) filler is known to be one of the most suitable fillers being used in epoxy-based foam composite because it has resistant to high temperatures, high strength-to-weight ratio, and has stable chemical compositions which can effectively reduce the density of the material as reported in our previous work [39]. Nanoclay was also selected to enhance the stiffness of the sandwich panel as it has proven to enhance stiffness of composite materials which was evident in our previous work, and also contributed to decrease in water absorption rate [39,40]. Therefore, combining these materials as a hybrid core can increase sandwich composite structures' bending stiffness and heat resistance which are very important for sandwich composite panels that find application in aerospace and other critical engineering applications. In recent times, polyvinyl chloride (PVC), polyurethane, wood, and polyethylene terephthalate (PET) have been utilized as cores [12,13,41], and syntactic foam, which has recently become the most widely utilized of all the cores [42,43]. Studying the tensile and flexural characteristics of sandwich composites consisting of a syntactic foam core and paper skin, it was found that, depending on the starch content of the glue used to join the paper skin and core, the skin paper increased the projected flexural strength by 40 % over syntactic foams [42]. Hybrid-filled foam panel made with HGM in an epoxy matrix has shown to be a suitable material with great structural strength due to its high specific stiffness and low density [39,44] and likewise, incorporation of nanoclay at the core or skin level of sandwich composite has been proven to enhance the toughness and impact performance of lightweight materials [40,45] because nanoclay has proven to be an effective filler with strong significant improvement in impact damage tolerance [46] and combining these fillers as the hybrid core will offer valuable insights into developing efficient strategies for fabrication of sandwich panels. Therefore, a sandwich composite structure developed with HGM and nanoclay as a hybrid core with banana fiber facesheets provides a novel approach to improving the structural, water uptake, buoyancy, and morphology behavior of sandwich composite panels because this area of study has not been fully explored.

The purpose of the study is to understand the influence of using an HGM/nanoclay hybrid core with a banana fiber facesheet on the mechanical properties of sandwich composite panels because using a natural fiber facesheet enhances the mechanical properties of sandwich structures. Hence, the mechanical properties of sandwich composite panels using hybrid cores were investigated in this research to understand the effect of combining hybrid core with banana fiber facesheet in sandwich composite materials. Tensile, flexural, impact, buoyancy, and water absorption properties of sandwich composite panels at various percentage loadings of hybrid core from 1 wt%HGM+1 wt%Clay up to 3 wt%HGM+5 wt%Clay of hybrid core were analyzed. The modified epoxy-based system is considered in this study to bond the banana fibers and the core to the facings forming a sandwich structure. These sandwich composite structures could show potential implications for a range of industries, including automotive, aerospace, and construction sectors, which are significant.

## 2 Methods and experimental details

### 2.1 Materials that were employed as constituents

Sandwich composites consist of both the core and the face sheets. In terms of the core materials, HGM-7019 with a density of 0.19 g/cm^3^ and Cloisite® 25A clay montmorillonite (MMT) with a density of 0.19 g/cm^3^ were used to create the hybrid core which was supplied by AMT Composites in South Africa and Southern Clay Products, Inc., USA. Banana fibers for facesheets (top and bottom facesheets) are obtained from Reddcolt Enterprises in India. Epoxy resin LR 30 and hardener LH 30 which are matrix materials were acquired from AMT composite Durban. The resin-to-hardener ratio remained constant at 10:2 according to the supplier's prescription and the density of resin is 1.13 g/cm^3^ while that of the hardener is 0.94 g/cm^3^. The unidirectional banana fibers utilized in this study were treated with 5 % NaOH and this was done to improve tensile strength and fiber-matrix adhesion due to the removal of both natural and manufactured contaminants and oils from the fiber's cell wall's exterior surface [[47], [48], [49]]. Table 1 displays the density values of the sandwich composite components.

**Table 1:** Properties of component materials used in sandwich composites.

Constituent Materials	Density(g/cm^3^)	Young's Modulus(GPa)	Poisson ratio
Banana fibre^x^	1.20	1.46	0.271
Matrix resin^y^	1.13	1.31	0.280
Sandwich composite^x^	1.157	1.63	0.276
HGM^y^	0.19	1.58	0.17
Clay^y^	0.19	2.65	0.14

x Calculated.

y As received from the supplier.

#### 2.1.1 Development of foam core

Our previous work provides detailed processing approaches for the core alone [39]. The hybrid core that was used is 3 mm thick.

### 2.2 Sandwich composite fabrication

**Fig. 1:**
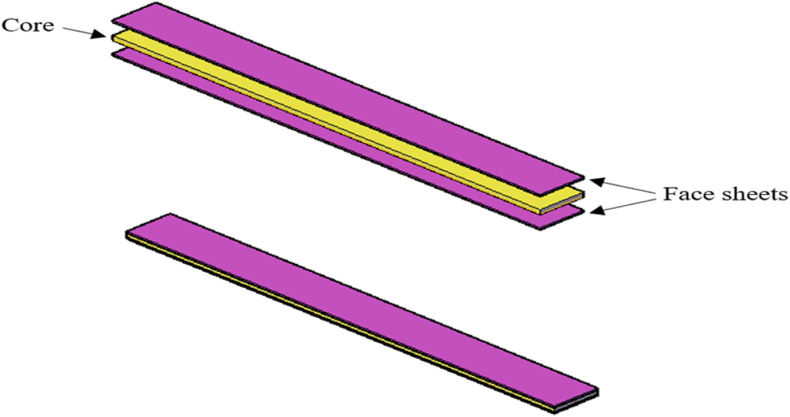
Schematic illustration of Sandwich Composites Panel.

The sandwich composite panels are made using a typical hand lay-up method, which is then followed by a moderate compression molding operation. It was made by placing the hybrid-filled core between the top and bottom facesheets of the sandwich composites as needed. By varying the volume fraction loading of fillers in the hybrid core, nine groups of sandwich composite samples were produced. Table 2 shows the identification of the hybrid core composition in sandwich composite while Table 3 shows the experimental density and theoretical densities of the sandwich composites developed for this study. Fig. 1 depicts a schematic representation of sandwich composite production. It was processed by placing the hybrid core between the sandwich composites’ top and bottom facesheets as needed. Banana fibers were prepared in a unidirectional method with (14.68g) by weight and 1.2 mm thickness each as top and lower facesheet. The size of the panel created for each sample was 300 ×140×5.4 mm^3^. The angle for overlaying the facesheet was 0^0^/90^0^ for all the sandwich composites. A ± 0.01 mm discrepancy in facesheet thickness was discovered, which can be explained by undulation during the fiber laying up process. For quick and easy removal of the sandwich composite material, wax was applied on the surface of the mold. During the preparation, care was taken to avoid the production of air bubbles. For efficient bonding, a 50 Kg load made of mild steel material was applied from the top, but before then a flat sheet plate made of neat epoxy material was placed on the panel for even distributions of resin in the facesheet of the sandwich panel, and the mold was then allowed to cure at room temperature for 48 h. After 48 h, the samples were removed from the mold and post-cured in a 70 °C oven for 4 h before cutting into the required size for tensile, flexural, impact, and water uptake tests with specifications according to their respective ASTM standards and tested accordingly. Each sandwich composite formulation was tested with three samples.

**Table 2:** Designation and detailed composition of the hybrid core.

Composite notation	Compositions
EPF	Filler(0 %) + Epoxy(100 %)
1HF	HGM(1 %) + Clay(0 %) + Epoxy(99 %)
1H1CF	HGM(1 %) + Clay(1 %) + Epoxy(98 %)
1H3CF	HGM(1 %) + Clay(3 %) + Epoxy(96 %)
1H5CF	HGM(1 %) + Clay(5 %) + Epoxy(94 %)
3HF	HGM(3 %) + Clay(0 %) + Epoxy(97 %)
3H1CF	HGM(3 %) + Clay(1 %) + Epoxy(96 %)
3H3CF	HGM(3 %) + Clay(3 %) + Epoxy(94 %)
3H5CF	HGM(3 %) + Clay(5 %) + Epoxy(92 %)

**Table 3:** The experimental density of sandwich composites with hybrid core.

Composite notation	Theoretical density	Experimental Density	Porosity (%)
	(g/cm^3^)	(g/cm^3^)	
EPF	1.157	0.997	3.797
1HF	1.102	0.994	9.800
1H1CF	1.091	0.987	9.532
1H3CF	1.032	0.946	8.333
1H5CF	0.978	0.916	6.339
3HF	1.060	0.962	9.245
3H1CF	1.032	0.981	4.942
3H3CF	0.978	0.903	7.669
3H5CF	0.931	0.880	5.477

### 2.3 Density

The theoretical density of sandwich composite materials expressed in terms of weight fractions of various constituents can be easily calculated using Agarwal's and Broutman Equation [50].

Density calculation.

The experimental densities of the sandwich composites were calculated using ASTM standard C271-94 [11]. The mixing rule(equation (1)) was used to determine theoretical densities, which were considerably greater than the experimental density [51]. Table 1 shows the densities of the component materials.

[1] $${ɕ}_{c}={ɕ}_{H}v_{H}+{ɕ}_{\text{cl}}v_{\text{cl}}+{ɕ}_{f}v_{f}+{ɕ}_{m}v_{m}$$

ɕ_c_ = density of the constituent, ɕ_f=_density of fibre

ɕ_H_ = density of HGM the, v_f_ = volume of fibre

v_H=_volume of HGM, ɕ_m_ = density of matrix

ɕ_cl=_density of clay, v_m=_volume of matrix

v_cl=_volume of clay.

The matrix void, which was computed as the variation between the theoretical and experimental densities, was reported in Table 3.

[2] $$\text{Pc}=\frac{{ɕ}_{th}-{ɕ}_{e}}{{ɕ}_{th}}\;X\;100$$

Where *Pc* is the sandwich composite porosity, ɕth is the theoretical density, and ɕ_e_ is the experimental density.

When estimating the sandwich composite density, all component material densities were taken into account.

The composition of the facesheet is uniform for all the sandwich panels, volume of fiber is 20%wt.Fibers while the volume of resin in each of the facesheet is 80%wt.Epoxy resin.

### 2.4 Tensile testing

**Fig. 2:**
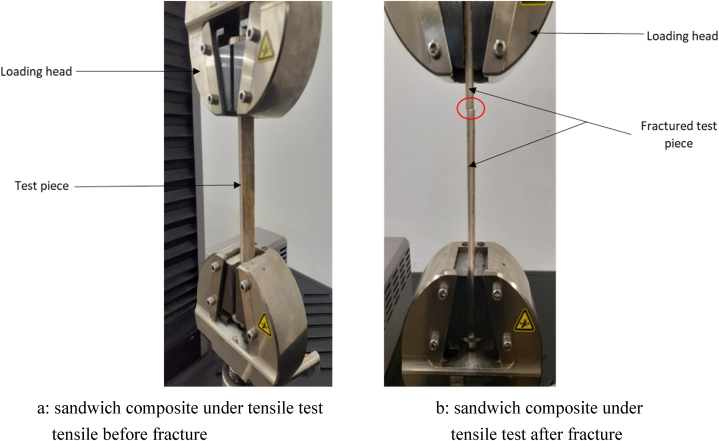
(a) sandwich composite under tensile test (b) sandwich composite under tensile before fracture tensile test after fracture.

Tensile testing is a common destructive mechanical testing technique used for composite materials. It helps determine how strong a material is and how much it can extend before failure. Yield strength, ultimate tensile strength, ductility, and Young's modulus of a material can be determined through tensile testing. Tensile testing was performed in the form of tension testing according to ASTM D3039 which can be seen in Fig. 2a and b, the standard test method for tensile properties of polymer matrix composite materials, at a test rate of 0.03 mm/s. The in-plane tensile characteristics of polymer matrix composite materials reinforced by high-modulus fibers are determined using this test method [52].

Samples for tensile and flexural properties were prepared following ASTM D 3039 test standard specifications on an MTS 793 machine that has a load cell of 30 KN using a constant testing cross-head speed of 2 mm/min. The tensile samples' length, width, and thickness were 250 mm × 25 mm × 5.4 mm. Four samples were tested from each foam panel sample, the mean values were employed in graphical representations and discussions.

The Young's modulus, *E* was taken on the linear slope of the curve, between two specific strains, Ɛ2 = 0.30 % and Ɛ1 = 0.15 %. The tensile strength, strain at break, and modulus of elasticity were determined from the following equations.

[3] $$\sigma =\frac{P}{A_{0}}$$

[4] $$Ɛ=\frac{\left(l-l_{0}\right)}{l_{0}}$$

[5] $$E=\frac{\left({\sigma \;}_{1}-{\sigma \;}_{2}\right)}{{Ɛ}_{2}-{Ɛ}_{1}}$$

Where;

***σ*** = tensile strength (MPa)

*P* = applied load (N)

*Ao* = original cross-sectional area of the sample in the gauge length (mm^2^)

Ɛ = Elongation

*lo* = original gauge length (mm)

*l* = instantaneous gauge length (mm) and

***σ***_1_, ***σ***_2_ = corresponding stress at the specific strain (MPa)

### 2.5 Impact test

**Fig. 3:**
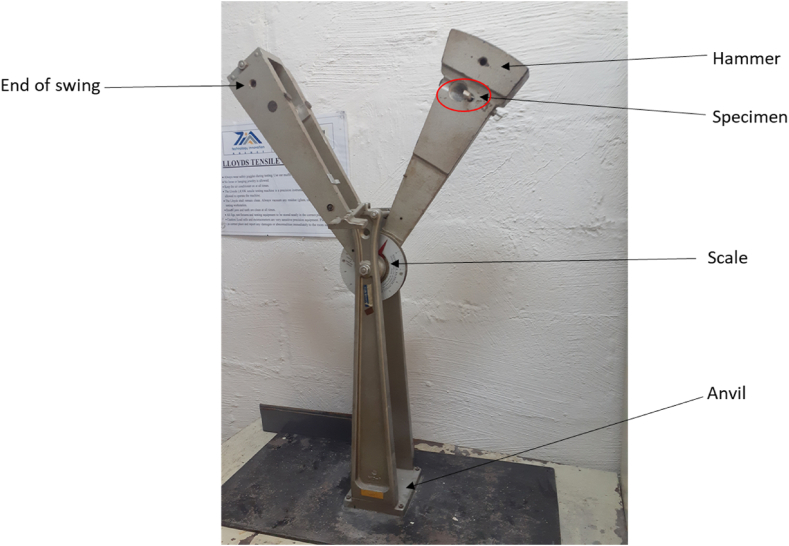
Sandwich composite under impact test.

Charpy is an industry-standardized high-strain rate test used to measure the amount of energy absorbed by a material during fracture. The energy absorbed by a conventional notched specimen while breaking under an impact load is measured in this test [53]. The test was carried out at room temperature as seen in Fig. 3 with a Tensometer Ltd., Croydon, England, Hounsfield Balance Impact Tester. The Hounsfield Balance Impact Machine has a three-point Charpy apparatus created in ASTM D6110-10 [40]. The geometric dimensions of the test specimens were 50 x 10 × 5.4 mm^3^ in length, width, and thickness cut from the foam panel. All impact samples had a 2 mm deep notch in the middle, opposing the impact area, with an 8 mm impact width. Three samples were tested for each volume fraction, and the average values collected were used to calculate energy absorption. The impact velocity was approximately 6.7 m/s. The average values collected were used to calculate energy absorption using the following equation.

[6] $$IS=\frac{\;AE\;}{\text{TW}}$$

IS: impact strength (kj/m^2^)

AE: absorbed energy (Joule)

T: specimen thickness (m)

W: Remaining width at notch (m)

### 2.6 Scanning electron microscope (SEM)

The SEM was used to examine and analyze the foam composite panel's fractured surface and microstructure. The fractured samples were viewed under SEM after being coated with gold to prevent charging. The microscopic images of all the fractured samples of sandwich composite materials were compared.

### 2.7 Flexural test

**Fig. 4:**
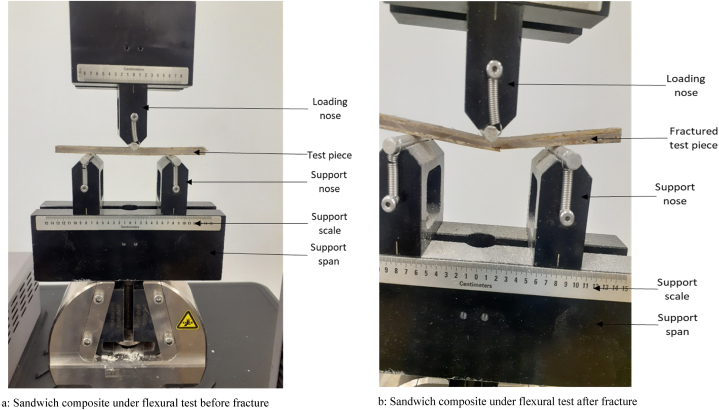
(a) Sandwich composite under flexural test before fracture (b) Sandwich composite under flexural test after fracture.

Three-point bending tests study the bending behavior of the core and sandwich structures. Tests were conducted according to ASTM C393, the standard test procedure for flexural properties of sandwich constructions at a test rate of 0.3 mm/min. This method was used to investigate the characteristics of flat sandwich structures exposed to flatwise flexure in such a way that the applied moments create bending of the sandwich-facing planes [54]. All tests were carried out at room temperature using MTS Criterion (Model 43), an electronic universal testing machine with a load cell capacity of 30 kN as seen in Fig. 4a and b. The bend fixture used was set to a span length of 80 mm with the nominal thickness of the core 3 mm, while the facesheet is 1.2 mm each at the upper and lower end with the width sustained at 14 mm. The samples were placed into the test fixture and loaded until failure occurred. Flexural stress and strain were calculated from the corresponding load–displacement data, to plot stress–strain curves. The sample deformation was photographed throughout testing.

Load–deflection data for each sample was collected and the maximum flexural stress, strain, and modulus were calculated using the following equations:

[7] $$\sigma_{f}=\frac{3PL}{{2bh}^{2}}$$

[8] $${Ɛ}_{f}=\frac{\;6Dd}{L^{2}}$$

[9] $$E_{f}=\frac{{\sigma f}_{2}-{\;\sigma f}_{1}}{{Ɛf}_{2}-\;{Ɛf}_{1}}$$

Where;

***σ****f* = stress at the outer surface at mid-span of the specimen (MPa)

*P* = load at a given point on the load-deflection point (N)

*L* = specimen length or support span (mm)

*b* = specimen width (mm)

ℎ = specimen thickness (mm)

Ɛ*f* = flexural strain in the outer surface (mm/mm)

*D* = maximum deflection of the center of the specimen (mm)

*d* = depth (mm) and.

*Ef* = flexural modulus (MPa)

### 2.8 Water absorption

Natural fibers are prone to water absorption due to their cellulose-rich chemical composition and hydrophilic nature. An increase in cellulose content tends to increase water absorption due to the increase in free hydroxyl groups within the fiber. Water absorption was measured according to ASTM C272, the standard test method for water absorption of core materials for sandwich constructions. This test method determines the relative amount of water absorption when immersed in water. It consists of exposing sandwich core specimens to a defined moisture condition and determining the amount of water absorbed by measuring the mass increase in the specimen. The dimensions of the water absorption samples are 100*25*5.4 mm. Equation (3) was used to calculate the increase in the percentage of water absorption rate.

$$\text{Increase}\;\text{in}\;\text{water}\;\text{absorption}\;\text{rate},\%=\frac{\;W-D}{D}$$

where W is the specimen weight after immersion (g) and D is the pre-immersion mass (g) [55].

### 2.9 Buoyancy

Buoyancy is a measure of the upward force exerted by a fluid against the weight of a partially or completely immersed item. The nine different compositions of sandwich composite samples prepared were submerged one after the other in a test tube containing water of 400 ml. For proper comparison, the weight of the submerged sandwich composites was the same. Three specimens each were submerged in the test tube filled with water. The buoyancy force is created by density differences in the gravitating fluid. The force of buoyancy is given by Equation 10

[10] Fb=Vo×D×Fg

Where Fb (N) = buoyancy force acting on the object.

Vo (m^3^) = volume of the submerged object.

D (Kg/m^3^) = density of the fluid the object is submerged.

Fg = force of gravity (N/Kg)

## 3 Discussion of the findings

### 3.1 Density of sandwich composites

The density of sandwich composite is determined by the relative quantity of matrix and reinforcing elements, and this is one of the very critical factors defining composites' qualities. The inclusion of voids in the sandwich composites accounts for the disparity in theoretical and experimental densities. As a result, determining the percentage of voids in the prepared samples of sandwich composites becomes critical. Table 3 shows the theoretical and experimental densities, as well as the volume proportion of voids in the sandwich composites with hybrid cores. Sandwich composite had more voids, which were attributed to poor filler bonding or fiber/matrix bonding and, ultimately to the epoxy matrix not completely wetting the banana fiber. The content of voids in sandwich composites is determined by the behavior of the hybrid cores with facesheet due to particle concentration of fillers in the hybrid core and also because natural fibers have lumens in their cellular structure that behave as voids, implying that such fiber naturally transports these spaces. Thus, it may contribute to an increase in void content in the sandwich composite [56].

### 3.2 Tensile properties of sandwich composites

**Fig. 5:**
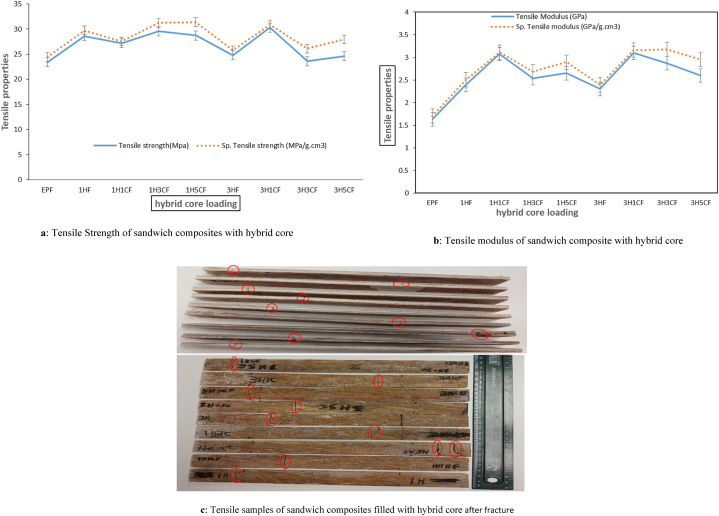
(a) Tensile Strength of sandwich composites with hybrid core (b) Tensile modulus of sandwich composite with hybrid core (c) Tensile samples of sandwich composites filled with hybrid core after fracture.

Determining the tensile properties is crucial because it offers details about different tensile characteristics (elastic limit, tensile strength, modulus of elasticity, etc.), and understanding how composite materials react to applied forces in tension is crucial because it offers details about the materials' ability to withstand tension. Since each composite has unique tensile characteristics, tensile tests are carried out to comprehend how various composites behave [57]. The tensile strength and specific tensile strength of sandwich composites are depicted in Fig. 2a with the effect of hybrid core loading on the tensile strength of sandwich composites. Varying tensile strength results were observed in the composites, this performance was a result of the number of voids observed in the sandwich composites due to the percentage of hybrid filler added in the core which is similar to results obtained by Basheer A. et al. [58] and also because natural fibers have lumens in their cellular structure that behave as voids, implying that such fiber naturally transports these spaces but the highest tensile strength was observed in 3H1CF which is 30.31 MPa with 3%HGM+1%Clay of hybrid core loading with banana fiber facesheet. This is because the fillers in the hybrid core provide reinforcement, allowing stress to flow from the matrix to the fillers. The sandwich composites containing 3H3CF hybrid core loading show a decrease in tensile properties which could be because of the filler packing or filler loading in the core as well as insufficiently rich epoxy areas. Furthermore, the possibility of fiber entanglements and filler agglomeration occurs in the sandwich composite, resulting in a reduction in stress transfer between the matrix and the fiber/filler. Similar results were reported by Boopaan et al. [59] for fiber-reinforced sandwich composites. Porosity and cavities affect the matrix-dominated characteristics like tensile, the flexural strength. Additionally, as shown in Fig. 5a and b, the density values of the sandwich composites were utilized to normalize the tensile strength and tensile modulus, producing specific tensile strength and specific tensile modulus. Due to the volume percentage of fillers employed in the construction of the hybrid core, this has little impact on the material. It was also observed that the specific tensile strength and specific tensile modulus are higher than the tensile strength and tensile modulus in all the composites because of their strength-to-weight ratio.

### 3.4 Flexural properties

**Fig. 6:**
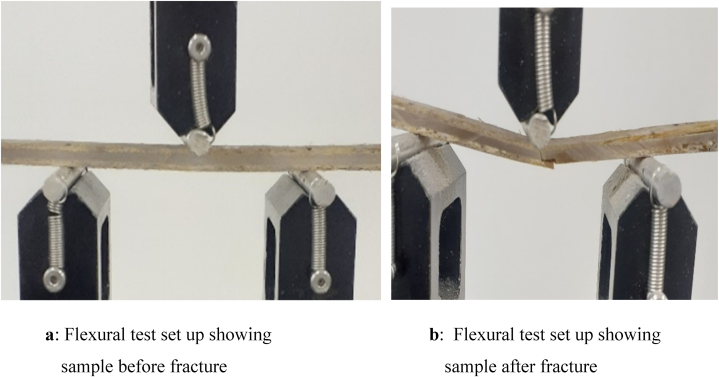
(a) Flexural test set up showing (b) Flexural test set up showing sample before fracture sample after fracture.

**Fig. 7:**
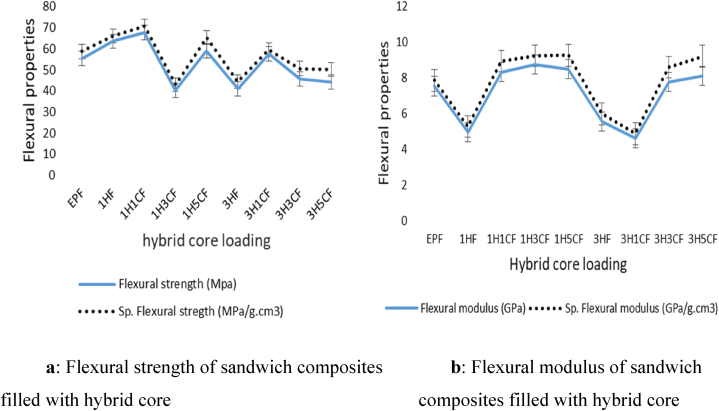
(a) Flexural strength of sandwich composites (b) Flexural modulus of sandwich filled with hybrid core composites filled with hybrid core.

Fig. 7a and b shows a summary of the flexural test 3-point bending findings which shows the graphs of the nine sandwich composite sequences. As can be seen in Fig. 7a and b, the flexural strength and flexural modulus were normalized using the sandwich composites' density values to get the specific flexural strength and specific flexural modulus. Due to the volume percentage of fillers employed in the construction of the hybrid core, this has little impact on the material. It was also observed that the specific flexural strength and specific flexural modulus are higher than the flexural strength and flexural modulus in all the composites because of their strength-to-weight ratio. Fig. 6 displays the average flexural performance of the sandwich structures, with the main form of failure observed at the lower face sheet undergoing tension as seen in Fig. 6b. It is worth noting that while the improved performance of sandwich structures with hybrid core may be due to the concentration of filler used in the core, the samples with hybrid core consist of slightly denser cores compared to the samples with virgin core as shown in Table 1. Fig. 7a compares the flexural strength of the nine sandwich composites, 67.79 MPa strength level at 1H1CF was observed and this represents the influence of the combined effect of hybrid core and banana fiber skin to increase strength. The differences in maximum flexural values are related to each sample's capacity to bear the bending force. A high degree of flexural strength at 1H1CF demonstrates the ability of the hybrid core in the sandwich structures to absorb more applied shear pressure than the HGM-filled core sandwich structure which is similar to work done by Pandey et al. [60]. This is due to the good bonding structure that exists between the facesheets and the hybrid core when the loads are carried together [61]. A significant correlation was detected between the strength values and the maximum flexural modulus, which was observed at 1H1CF. This suggests a strong link between the face sheets and the core. The rising sequence for flexural strength and modulus is > 1H1CF > 1HF > 1H5CF > 3H1CF > EPF>3H3CF > 3H5CF > 1H3CF, with a percentage increase of over 18.11 % and 16.07 % in terms of flexural strength and modulus, respectively. It was also determined that the bending strength exceeded the tensile strength, which corresponds to the Leguillon et al. [62] investigations when they stated that the bending strength of the sandwich composite panel was larger than the tensile strength. Overall, the sandwich composite structures using hybrid cores demonstrated better improvement in flexural properties, with 1H1CF showing an average flexural strength of 67.80 MPa, and 1H3CF showing a flexural modulus of 8.74 GPa.

### 3.5 Impact properties of sandwich composites

**Fig. 8:**
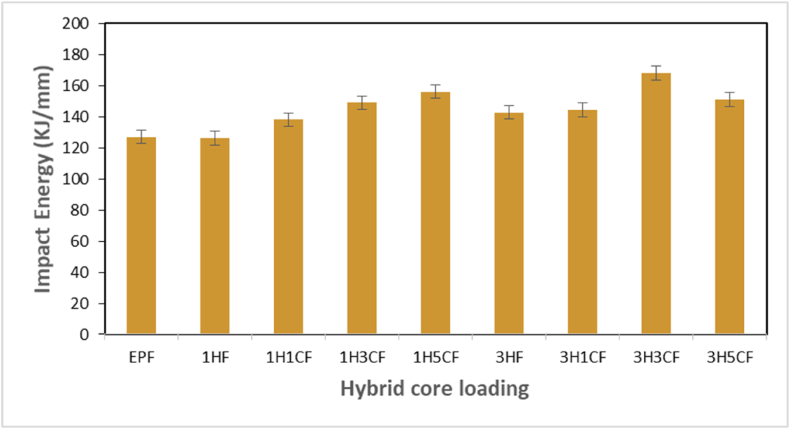
Impact strength of sandwich composites with hybrid core.

Impact tests are used to measure a material's toughness. The toughness of a material is a measure of its capacity to absorb energy during plastic deformation. Fig. 8 depicts the effects of filler/fiber loading on sandwich composites' impact energy. The impact energy of the sandwich composites filled with HGM core is found to be lesser than the sandwich composite panels filled with the hybrid core. Fractures in the specimen begin with crack propagation caused by a lack of a high level of adhesion between the facesheets and the core, following that, fiber breakage, matrix fracture, and pull-out ensue. The findings of the experiments revealed that using a hybrid core in sandwich composite material increases the bonding capability and this results in increased impact strength. The greatest amount of impact energy value of 168.1 KJ was achieved for the sandwich composite at 3H3CF of hybrid core loading. A similar pattern of increasing impact strength value with increasing filler/fiber loading was also reported by Cabral et al. and Eyvazian et al. in their studies [[63], [64], [65]]. The increase in impact strength with increasing filler concentration in hybrid core loading could be attributed to more energy being required to break the connection between the interlaced filler/fiber bundles [66].

### 3.6 Surface morphology

**Fig. 9:**
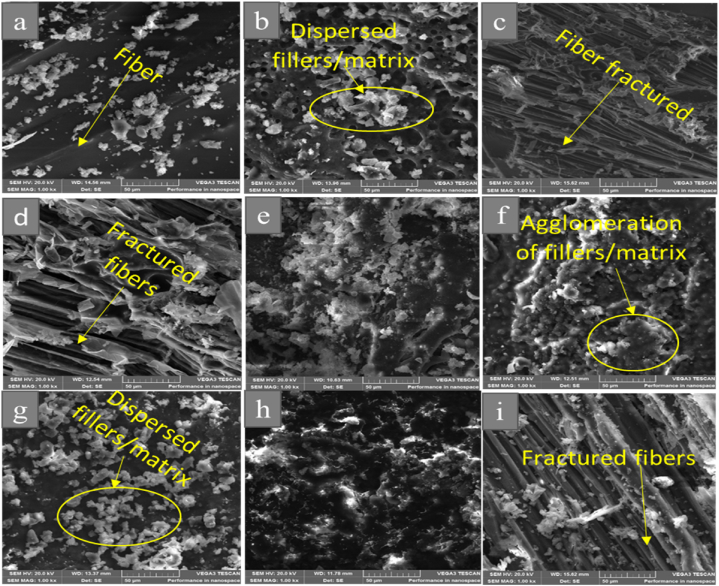
SEM image of the tensile fracture surface of Sandwich composite for (a) EPF (b) 1HF (c) 1H1CF (d) 1H3CF (e) 1H5CF (f) 3HF (g) 3H1CF (h) 3H3CF (i) 3H5CF.

Following the tensile test, the surface properties of the sandwich composite material were investigated using a scanning electron microscope (SEM). Fig. 9a-i shows a micrograph of a tensile test broken specimen of the sandwich composite materials. Using scanning electron microscope (SEM), the microstructural pattern of the tensile cracked specimens on each composite during testing was characterized for sandwich composite materials with different hybrid core formulations. The shattered fibers and distributed fillers in Fig. 9b, d, and 9g exhibit improved load-bearing and reduced agglomeration as a result of the strong link between the facesheets and hybrid core. The sandwich composites' strength and modulus improved as a result. Clustered fillers with a rough surface that led to early breakage and low strength values were seen in Fig. 9e, f, and 9h. This might occur as a result of their independent load-bearing before the core fracture [67]. Additionally, Fig. 9c and i showed the tensile cracked fibers, indicating increased load bearing because of the strong link between the hybrid core and facesheets. As a result, the sandwich composites experienced an improvement in modulus and strength.

### 3.7 Water absorption behavior of sandwich composites

**Fig. 10:**
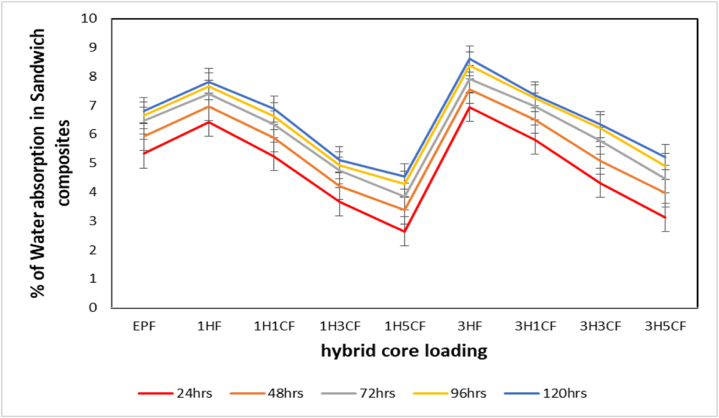
Percentage of Water Absorption of sandwich composites with hybrid core.

Fig. 10 depicts the ability of sandwich composites to absorb water as a function of immersion time. It displays the percentage of water absorption over hours. It can be seen that the absorption behavior steadily increases with immersion duration for roughly 6 days. From the graph, it is clear that the sandwich composites with HGM cores absorb water at a considerably higher rate than the sandwich composite with hybrid cores which is because water invades the interface layer of HGM and epoxy resin of HGM and the pores, this may cause damage to the HGM at higher concentration. A maximum of 4.54 % absorption is measured in the sandwich composites with the hybrid core while 8.61 % is observed in the sandwich composites with HGM core due to the presence of nanoclay in the hybrid core which provides moisture resistance to the sandwich composites because clay contains silicon-oxygen (SiO_4_) tetrahedron bilayers with deep-rooted octahedral layers of aluminum and iron which can resist moisture and this is similar to result reported by Nemati Giv et al. [68] on water absorption behavior of composites filled with nanoclay.

### 3.8 Water contact angle

**Fig. 11:**
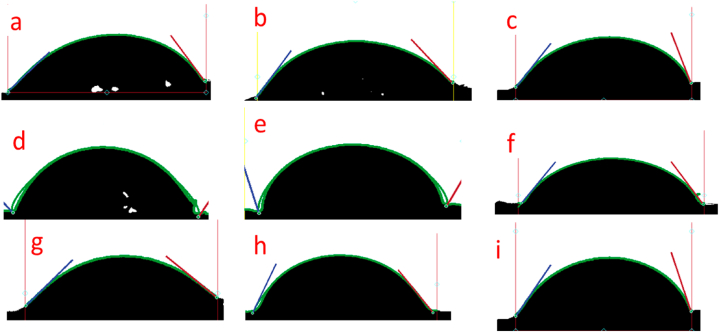
Images of water contact angles of (a) EPF (b) 1HF (c)1H1CF (d)1H3CF (e) 1H5CF (f) 3HF (g) 3H1CF (h) 3H3CF (i) 3H5CF.

The contact angle test was used to analyze the wettability behavior of the sandwich composite materials filled with the hybrid core. The contact angle of water on the transverse surface of sandwich composite panels is summarized in Table 4. According to Klein N S et al. and Xiong B et al. [69,70], a surface is considered hydrophilic when the static water contact angle θ is less than 90^0^ and hydrophobic when θ is greater than 90^0^. In this study, all the sandwich composite samples exhibited hydrophilic behavior, indicating their affinity for water because of their natural use as the facesheet. However, the study revealed that sandwich composite 1H5CF with the highest concentration of nanoclay displays an average contact angle of 62.13 indicating a significant change in the hydrophilic behavior of the sandwich composite panel when compared to other samples of sandwich composite as seen in Fig. 11. This behavior justifies the reason why the sandwich composite sample 1H5CF exhibits the lowest percentage of water absorption in Fig. 7 due to the concentration of nanoclay in the hybrid core.

**Table 4:** Water contact angles of sandwich composite panels.

Materials	Water contact angles(WCA) θ^0^
EPF	47.41 ± 0.43
1HF	37.55 ± 0.39
1H1CF	45.17 ± 2.13
1H3CF	58.22 ± 0.87
1H5CF	62.03 ± 0.34
3HF	33.87 ± 1.85
3H1CF	41.47 ± 3.31
3H3CF	52.74 ± 0.63
3H5CF	56.67 ± 2.74

### 3.9 Buoyancy behaviour of sandwich composites

**Fig. 12:**
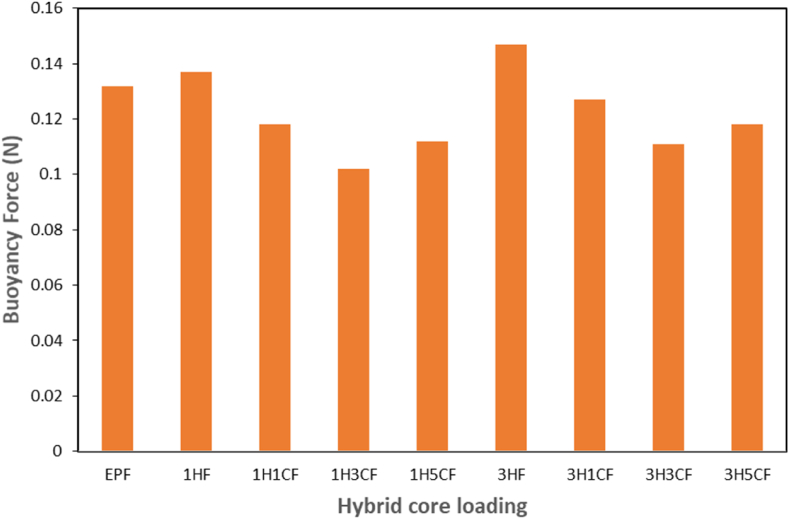
Buoyancy Force (N) of hybrid-filled Sandwich Composites.

Fig. 12 shows the buoyancy force (Fb) of the Sandwich composites. The 3HF had the highest Fb of 0.147 N, while the 1H3CF had the lowest Fb of 0.102 N. The maximum Fb found at 3HF could be because of its higher concentration of HGM than all other sandwich composites which could indicate that when submerged in fluid, with more Fb for an up-thrust to float than other sandwich composites filled with hybrid core makes it have better buoyancy force than other sandwich composite. Furthermore, because of a high percentage of cellulose in banana fibers which was used as a face sheet, and concentration of nanoclay in the core, 1H3CF will sink faster when submerged but when the hybrid core with a higher concentration of HGM is introduced into the sandwich composite, it can reduce the water absorption of the sandwich composites due to the low density of the fillers in the hybrid core.

### 3.10 Strength-to-weight ratio

The strength-to-weight ratio of the hybrid core refers to the relationship between the weight of a sandwich composite structure and the amount of weight it can support before collapsing. Sandwich composite structures are known for having an exceptional strength-to-weight ratio, due to their density, which provides a balance between structural integrity and material usage. It was observed that the use of HGM and nanoclay as hybrid core enhanced the sandwich constructions' strength-to-weight ratio in tensile strength and flexural strength. Strength-to-weight characteristics of the sandwich structures, as seen in Fig. 5, Fig. 7, undergoing tensile and flexural have improved with the addition of a hybrid core in the sandwich respectively.

## 4 Conclusion

In conclusion, the study demonstrates the feasibility of using hybrid core materials with banana fiber facesheet in a sandwich composite structure. The use of natural fiber at the facesheet provides a sustainable alternative to traditional structures made of non-environmentally friendly materials. The combined effect of HGM and nanoclays in the hybrid core enhanced the tensile strength, flexural strength, and impact resistance of the structures while reducing water uptake. The combined effect of HGM and nanoclay in the hybrid core enhanced the flexural strength and tensile strength by 22.11 % and 29.53 %, respectively. The sandwich composite structures with 3H3CF absorbed 32.26 % more impact energy than other remaining samples with different hybrid core loading. The sandwich composite structure with a hybrid core showed improved tensile strength while reducing moisture uptake, making them a promising option for use in various applications, including aerospace, automotive, and marine industries. The sandwich structure with hybrid core showed a tensile strength that was 6.05 % higher than those sandwich composites using solely HGM core and was found to be hydrophobic, leading to a reduction of 8.61 % in water uptake compared to solely HGM core and this is significant because it gives the basis for comparison between sandwich panels with hybrid fillers in the core and sandwich panels with only HGM filler in the core. In summary, this study highlights the potential of using banana fibre as a facesheet with a hybrid core for sandwich composite offers desirable properties while being environmentally friendly, providing a valuable contribution to the growing body of research in sustainable materials for sandwich composite structures.

## Funding Information

Research and Postgraduate support office, Durban University of Technology.

## CRediT authorship contribution statement

**Ayodele Abraham Ajayi:** Writing – review & editing, Writing – original draft, Methodology, Investigation, Formal analysis, Data curation, Conceptualization. **Mohan Turup Pandurangan:** Validation, Supervision. **Kanny Krishnan:** Project administration.

## Declaration of competing interest

The authors declare that they have no known competing financial interests or personal relationships that could have appeared to influence the work reported in this paper.
